# Cotyledonoid dissecting leiomyoma of the uterus: a case report

**DOI:** 10.1186/1746-1596-2-18

**Published:** 2007-06-13

**Authors:** Annikka Weissferdt, Madhavi B Maheshwari, Gabrielle P Downey, Terence P Rollason, Raji Ganesan

**Affiliations:** 1 Department of Histopathology, Birmingham Women's Hospital, Metchley Park Road, Edgbaston, B15 2TG, UK; 2 Department of Histopathology, Sandwell and West Birmingham Hospitals, Dudley Road, Birmingham, B18 7QH, UK; 3 Department of Obstectrics and Gynaecology, Sandwell and West Birmingham Hospitals, Dudley Road, Birmingham, B18 7QH, UK

## Abstract

Cotyledonoid dissecting leiomyoma of the uterus is a recently described rare variant of benign uterine leiomyoma. We report a case of cotyledonoid dissecting leiomyoma in a 52 year old woman who presented with menorrhagia and abdominal pain. An ultrasound scan showed a bulky uterus and a cystic heterogenous mass near the left ovary. At hysterectomy, the left broad ligament mass was removed. This was continuous with an ill-defined nodular area in the myometrial fundus. Microscopy revealed a benign smooth muscle proliferation in the myometrium that extended beyond the uterus and into the broad ligament. The lesion appeared to be dissecting the myometrial fibres and showed areas of oedema, hyalinisation and perinodular hydropic change. Cellular atypia, mitoses and coagulative necrosis were absent. The patient is alive and well 18 months after surgery. It is important to recognize this benign and unusual appearing variant of leiomyoma in order to prevent inappropriate treatment.

## Background

Uterine leiomyomas can present in a variety of unusual growth patterns. One of the most unusual variants is called cotyledonoid dissecting leiomyoma or Sternberg tumour [1]. These tumours have been described to occur in women of the reproductive age group [1]. They tend to arise in the subserosal myometrium of the lateral aspect of the uterus especially the uterine cornu and extend into the broad ligament and pelvic cavity. The exophytic component has characteristic spongy, bulbous protuberances over the surface that appear congested and red, resembling placental tissue. The intramural component dissects into the fascicles of the myometrium in an irregular fashion. There usually is extensive hydropic degeneration resulting in the characteristic pattern of perinodular hydropic degeneration. Cellular atypia, mitoses and coagulative tumour necrosis are typically absent [1]. There often is confusion between this lesion and smooth muscle tumours that have somewhat similar pathologic features eg. intravascular leiomyomatosis. In addition, the worrying appearances of the gross specimen are often mistaken for malignant or non-uterine lesions that may result in overtreatment. To date, no malignant behaviour or recurrence has been described in these lesions with the longest follow-up period amounting to 41 years [1].

## Case report

A 52 year old woman, gravida 5, para 5, presented with a 5 months history of menorrhagia, abdominal pain and tiredness. A transvaginal ultrasound scan showed a bulky uterus with an irregular and thickened endometrium of 14 mm. A 10 mm well rounded and echo poor area was also noted in the endometrium. In the region of the left ovary there was a cystic lesion that had thick irregular borders and solid components measuring 40 mm in maximum dimension. The right ovary was unremarkable. The CA 125 level was 12 units/ml. Since the left ovarian lesion was reported as suspicious the patient underwent surgery. At the time of laparotomy, a bulky uterus was seen and a spongy lesion was noted in the region of the left ovary. A total abdominal hysterectomy with left salpingo-oophorectomy was performed and the patient is alive and well 18 months after the operation.

## Methods

Tissue sections were fixed in 10% formalin, embedded in paraffin and stained with haematoxylin-eosin. Immunohistochemical staining with the streptavidin-biotin peroxidase detection system was performed using a Ventana automated immunostainer (Ventana, Tucson, Arizona). The antibodies smooth muscle actin (1A4, 1:400, Dakopatts, Glostrup, Denmark), desmin (D33, 1:100, Dakopatts, Glostrup, Denmark), CD10 (56C6, NEAT (Ventana kit), Ventana, Tucson, Arizona), HMB45 (HMB45, 1:50, Dakopatts, Glostrup, Denmark) and CD31 (1A1O, 1:40, Novocastra, Newcastle upon Tyne, UK) were used.

## Results

**Figure 1:**
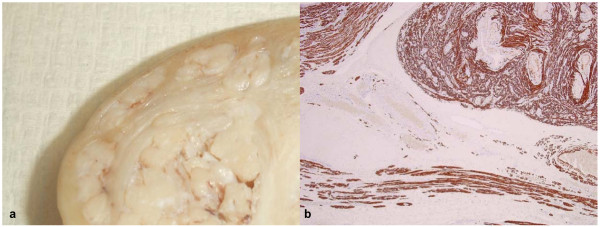
a Macroscopic appearance of the lesion mimicking an invasive tumour. b (desmin, × 40): Immunohistochemistry shows both the lesion and the surrounding myometrium are desmin positive.

Macroscopic examination of the specimen revealed a corpus and cervix 110 × 60 × 50 mm with an attached left fallopian tube 30 mm in length and left ovary 20 × 15 × 10 mm. A large mass was present in the left broad ligament in the region of the left ovary measuring 60 × 40 × 30 mm. This mass had a red, spongy, cystic and gelatinous cut surface reminiscent of placental tissue and was distinct from the ovary and fallopian tube but apparently continuous with an ill-defined nodular area in the myometrium of the uterine fundus (Fig. 1a). This nodular area also appeared to be continuous with a submucosal leiomyoma measuring 20 × 20 × 10 mm.

**Figure 2:**
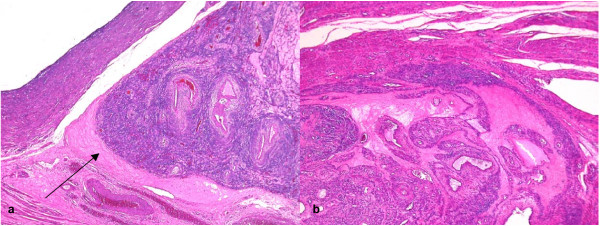
a (haematoxylin-eosin, × 40): The leiomyoma dissects into the surrounding myometrium (black arrow) at the cornu. b (haematoxylin-eosin, × 40): There is hydropic change around groups of smooth muscle bundles – a pattern referred to as perinodular hydropic degeneration.

Histological examination showed that the submucosal leiomyoma was composed of bland smooth muscle cells growing in broad, sweeping fascicles with no cellular atypia, abnormal cellularity or increased mitotic activity (up to 1 mitosis per 10 high power fields). Immunohistochemically, the constituent cells were positive with smooth muscle actin and desmin (Fig. 1b) while CD10, CD31 and HMB45 were negative. This leiomyoma was continuous with the ill-defined nodular area identified macroscopically. Here, the tumour cells were noted to grow into the surrounding myometrium in a dissecting pattern (Fig. 2a). Furthermore, prominent hydropic changes amounting to perinodular hydropic degeneration were present (Fig. 2b) and the lesion extended beyond the confines of the uterus and into the broad ligament where these features were exaggerated. The fascicles of muscle appeared disorganized but there was no evidence of coagulative necrosis. Multiple congested blood vessels were present throughout the lesion but there was no intravascular growth. The fallopian tube, ovary, endometrium and cervix were unremarkable.

## Discussion

Uterine smooth muscle tumours are known to exhibit a wide variety of growth patterns. One unusual variant is the cotyledonoid dissecting leiomyoma of the uterus or "Sternberg tumour" first described in 1996 by Roth et al. [1]. Including our current case, only 10 cases have been described in the English literature to date [1-6]. The case presented here demonstrated the typical features described for these tumours: an exophytic component of bulbous (cotyledonoid) smooth muscle grossly resembling placental tissue protruding from the uterine surface near the region of the cornu and extending into the the broad ligament. This exophytic mass was in continuity with an intramural component dissecting the surrounding myometrium in a sinuous pattern. The neoplastic smooth muscle cells formed disorganized fascicles in contrast to the organized pattern seen in conventional leiomyoma but did not show any evidence of nuclear atypia, increased mitotic activity or coagulative tumour necrosis. Areas of oedema and perinodular hydropic degeneration were present. The lesion was highly vascular containing multiple congested blood vessels but intravascular growth was absent.

Description of new variants of any lesion comes with attendant issues regarding nomenclature. Apart from the cotyledonoid dissecting leiomyoma described by Roth et al. [1] there are closely related variants demonstrating similar pathologic features. This similarity is mirrored in the terminology. A variant called cotyledonoid leiomyoma was described by Roth and Reed [7] in 2000. This lesion resembled cotyledonoid dissecting leiomyoma but lacked a parent intramural component. The same authors described another related variant called intramural dissecting leiomyoma [8]. This lesion shows intramural dissection but lacks the extrauterine component seen in cotyledonoid dissecting leiomyoma. Another smooth muscle tumour was described showing the characteristics of cotyledonoid dissecting leiomyoma with features of intravascular leiomyomatosis [9]. It was proposed that this variant is called "cotyledonoid hydropic intravenous leiomyomatosis". Whereas cotyledonoid dissecting leiomyoma is a benign lesion that has shown no malignant behaviour or recurrence on follow up [1], the prognosis for intravenous leiomyomatosis is different. Although intravenous leiomyomatosis is also regarded as a benign variant of smooth muscle tumour, the vascular invasion may extend as far as the extrauterine pelvic veins, the inferior vena cava and even the right heart [10-12]. Pulmonary metastases have also been reported [13] and the rate of recurrence and progressive growth is higher than in typical leiomyoma [14].

Therefore, care should be taken not to confuse these new variants of smooth muscle tumours with unusual growth patterns. This is especially important for tumours with different prognostic implications like the proposed "cotyledonoid hydropic intravenous leiomyomatosis" [9]. Careful assessment of the pathologic features will be necessary to prevent confusion and resulting misdiagnosis.

In summary, cotyledonoid dissecting leiomyoma is a rare variant of benign leiomyoma that is closely related to and shares some features with other and similarly named leiomyoma variants. Not only can its bizarre gross appearance lead to a mistaken diagnosis of a malignant neoplasm, but it should also be distinguished from other unusual leiomyoma variants, especially to exclude intravascular spread, for appropriate classification and potential prognostic implications.

## Competing interests

The author(s) declare that they have no competing interests.

## Authors' contributions

AW drafted the manuscript. RG and TR helped to draft the manuscript. MM and GD contributed the clinical background.

All authors read and approved the final manuscript.
